# Wakayama symposium: interface between innate and adaptive immunity in dry eye disease

**DOI:** 10.1186/s12886-015-0133-9

**Published:** 2015-12-17

**Authors:** Kyung-Sun Na, Kyu-Yeon Hwang, Hyun-Soo Lee, So-Hyang Chung, Jee Won Mok, Choun-Ki Joo

**Affiliations:** Department of Ophthalmology, Yeouido St. Mary’s Hospital, College of Medicine, The Catholic University of Korea, Seoul, South Korea; Department of Ophthalmology, Konyang University Hospital, Daejeon, South Korea; Department of Ophthalmology and Visual Science, Seoul St. Mary’s Hospital, College of Medicine, The Catholic University of Korea, Seoul, South Korea; Catholic Institutes of Visual Science, The Catholic University of Korea, Seoul, South Korea

**Keywords:** Dry eye disease, Innate immunity, Adaptive immunity, Transition

## Abstract

Although the mechanism of dry eye disease is not clearly understood, it is certain that inflammation and the immune response play a major role in determining the health of the ocular surface in dry eye patients. Accurate ocular surface characterization during the early stages of dry eye disease is critical for successful treatment, because there exists no single standard, objective test to diagnose the early phase of dry eye disease. The treatment target should be direct to prevent the perpetuation of chronic inflammation and immune responses. Numerous studies have categorized dry eye disease as an autoimmune-related inflammatory disease. However, relatively little is known about how innate immune mechanisms act following a local insult, why some patients are particularly vulnerable, and why local inflammation fails to resolve in these patients. Within this review, particular attention will be given to the very early events and corresponding defense mechanism in dry eye disease. The transition from innate to adaptive immunity will also be discussed.

## Background

The tear film, lacrimal glands, corneal and conjunctival epithelia, and meibomian glands constitute a lacrimal function unit (LFU) that serves to preserve the health of the ocular surface. The components of the LFU work together to maintain homeostasis despite internal and external insults [1, 2]. Dry eye disease (DED) is a highly prevalent inflammatory disease of the LFU that is multifactorial in nature. The definition of DED has evolved from mere tear deficiency to chronic inflammation and the resultant immunologic responses [3, 4]. Whether the inflammation is a cause or result of DED remains to be elucidated. The mechanisms explaining how and when homeostasis is disrupted following local insult or inflammation of the ocular surface are unclear.

The immune reaction comprises innate and adapted immunity, which differ vastly in terms of methodology as well as objective. Recent research has examined how the innate immune system influences adaptive immune responses [5]. Understanding the interface between innate and adaptive immunity would help to define the factors that trigger the adaptive immune response and allow for analysis of autoimmune and allergic diseases from a new perspective. Although the immunopathologic events that sustain the systemic adaptive immune response in DED have been characterized, the stressor that triggers the innate immune response and the interface between innate and adaptive immune mechanisms are not well defined. If the complicated crosstalk between innate sensory function and the adaptive response at the ocular surface could be understood, the pathogenesis of DED would be clearer. We aimed to briefly review the clues of innate immunity and the interface between innate and adaptive immunity in DED.

### Innate immunity

In DED, a chronic inflammatory reaction is generated at the ocular surface, accompanied by the destruction of epithelial tight junctions and finally sloughing of the surface epithelia [6]. The epithelium is part of the innate immune response, playing a crucial role in preventing the invasion of ocular tissue by foreign bodies or microorganisms [7]. Eyelid blinking, the barrier posed by the epithelium, secretory proteins such as lysozyme, and conjunctival mucous are additional aspects of the innate immune response at the ocular surface [8].

Corneal and conjunctival epithelial and epithelial-associated Langerhans cells are known to express a range of both toll-like receptors (TLR) and NOD-like receptors (NLR). The TLR and NLR pathways are the primary route by which host cells detect the presence of foreign invaders. The responses triggered include the production of cytokines, chemokines, and antimicrobial peptides. Increasing the expression of TLR or NLR may prevent the risk of infection but may also lead to inflammation. There is some evidence that TLR expression is modulated at the ocular surface in DED. In an experimental DED model, TLR2-4 and TLR9 expression was increased at the ocular surface and lacrimal glands [9, 10].

The rate of epithelial turnover is increased in DED, with viable corneal surface cells shed by classical apotosis [11, 12]. Dead cells can release endogenous extracellular DNA (eDNA), a type of damage-associated molecular pattern (DAMP) that activates innate immunity [13]. Sonawane et al. found that eDNA and neutrophils were present on the ocular surface in DED patients and suggested that eDNA production and clearance mechanisms are dysregulated in DED [14]. The authors suggested that accumulated eDNA and neutrophil extracellular trap (NET) in the pre-corneal tear film would result in the inflammation characteristic of DED.

Lactoferrin, which is secreted by acinar cells of the lacrimal glands, has anti-inflammatory, anti-cancer, and immune-modulating properties. Lysozyme, another secretory protein from acinar cells in the main lacrimal gland and conjunctival accessory lacrimal glands, attacks the cell walls of bacteria. The levels of lactoferrin and lysozyme are reported to be decreased in the tear fluid of DED patients [15, 16]. The level of secretory IgA (sIgA), a key factor that protects against microbes in the mucosa, is decreased in DED patients [16]. Thus, decreased sIgA levels inhibit proper modulation of the ocular surface flora, which in turn increases the risk of microbial infection. The expression of secretory phospholipase A2 (sPLA2) is increased in the tears of DED patients, which suggests that sPLA2 plays a major role in preventing microbial infection at the ocular surface in DED patients [17]. The altered expression of molecules involved in the innate immune response in dry eye may have other effects on the innate immune system as well as trigger an adaptive response if not properly managed.

Mucins keep the ocular surface moist and protect it from external stimuli, and the induction of mucin from the ocular surface may facilitate the stability of the tear film [18]. Mucins are high-molecular weight glycoproteins characterized by their extensive O-glycosylation [19]. Major mucins expressed by the ocular surface epithelia include cell surface-associated mucins MUC1, MUC4, MUC16, and the gel-forming mucin MUC5AC. Recent advances using functional assays have allowed the examination of their roles in the protection of corneal and conjunctival epithelia. Among these, MUC1 genotype polymorphism has been shown in DED [20]. Differences in MUC1 genotypes between healthy controls and DED patients could explain the loss of ocular surface integrity. The loss of mucins at the ocular surface may result in an increased risk of infection and epithelial stress, which could trigger the adaptive immune response.

Very recently, Desiccating stress-induced chemokine expression in the epithelium was shown to be dependent on upregulation of NKG2D/RAE-1 and release of IFN-γ in experimental DED [21]. Upregulation of CXCL9, CXCL10, and CXCL11 expression was notedto be T cell–independent, requiring IFN-γ–producing NKG2D^+^ NK cells that are activated in response to DS-induced stress signals. This suggest that the triggering immune response in DED pathology.

### Triggering adaptive immune response

Environmental triggers may also contribute to the initiation and perpetuation of the disease. The activation of innate immunity results in antigen presentation, T-cell activation, and the release of pro-inflammatory cytokines [22]. These changes activate the adaptive immune system and auto-antibodies. No known antibodies are known to be involved in DED; however, there is mounting evidence that DED is a localized self-antigen–driven autoimmune-based inflammatory disease [4].

Immune-mediated inflammatory disease (IMID) is associated with various conditions. While the exact etiology is undefined, both genetic and environmental factors play an important role in its pathogenesis. Recently, genome-wide association studies (GWASs) using millions of nucleotide polymorphisms as genomic markers and non-synonymous single nucleotide polymorphisms (nsSNP) have identified loci involved in IMID susceptibility [23]. Murakami et al. also reported that IL1B polymorphisms (genotypes CC, TT, and AA in positions -511, -31, and 3877, respectively) were significantly less frequent in Sjogren’s syndrome patients than controls or patients with SLE [24]. We identified certain genetic variations by screening for pro-inflammatory cytokine genes in Korean non-Sjogren’s DED patients. Among the screened genes, rs1143634 of interleukin-1 beta (IL1B) and rs8192284 of interleukin-6 receptor (IL6R) were linked to susceptibility among Korean non-Sjogren’s DED patients [25]. IL1B is a pro-inflammatory cytokine that is considered to be important in the pathogenesis of DED [26]. We also observed that the genotypic and allelic distributions of rs8192284 in the IL6R gene differed between DED patients and controls. The exact role of IL6 is unclear, and both its pro- and anti-inflammatory properties require further investigation. The balance between pro- and anti-inflammatory effects of IL6 was previously suggested to favor pro-inflammatory molecules under various environmental conditions [27].

The inflammatory response can resolve insults to the tissue and restore homeostasis [5]. However, inappropriate control of the natural defense mechanism results in chronic inflammation and disease progression. The resolution of inflammation is critical to the transition from innate to adaptive immunity [5, 28]. The inflammatory IL6 and soluble IL6 receptors are known to direct the transition from innate to adaptive immunity [28]. Traditionally, IL6 was regarded as an activator of acute-phase responses; recent findings have shown a series of IL6-mediated events are critical for resolving the innate immune response [29]. Levels of IL6, which may contribute to DED pathogenesis, are reported to be increased in the tears and conjunctival epithelium of DED patients [30]. The expression of soluble IL6 receptor (sIL-6R) in tears is upregulated in chronic inflammatory conditions affecting the ocular surface [31, 32]. Previously, we demonstrated that the levels of IL-1B, IL-6 and their soluble receptors sIL-1R1 and sIL-6R were significantly elevated in the tears of patients with DED [33]. We assumed that the IL-6/sIL-6R complex mediates the pathogenesis of DED and that the marked elevation of its natural antagonist, soluble glycoprotein 130 (Sgp130), may act to preserve ocular homeostasis in response to local inflammation. Interestingly, we also demonstrated decreased levels of IL-17A and interferon gamma (INF-r) in the tears of DED patients. We interpreted the results as evidence that the suppressor T-cell, which is a natural inhibitor of self-reactive Th1 (INF-r), Th2 (IL4+), and Th17(IL17A) cells, may function to some extent in the mild and early stages of DED [34]. In the clinical setting, it is rare to see advanced DED unaccompanied by systemic autoimmune disease. Moreover, patching the eyelid to protect against external stimuli and supplying artificial tears without any anti-inflammatory agent can resolve the symptoms and signs of mild DED. This phenomenon suggests that normal tissue architecture is preserved despite local insult and inflammation. However, when some mediators such as sIL-6 act negatively, various cytokines and chemokines act on both arms of the immunologic response. The interface between the innate and adaptive immune responses, and triggering factors initiating adaptive immune responses in DED remains to be elucidated by future clinical trials and research approaches.

## Discussion

**Fig. 1:**
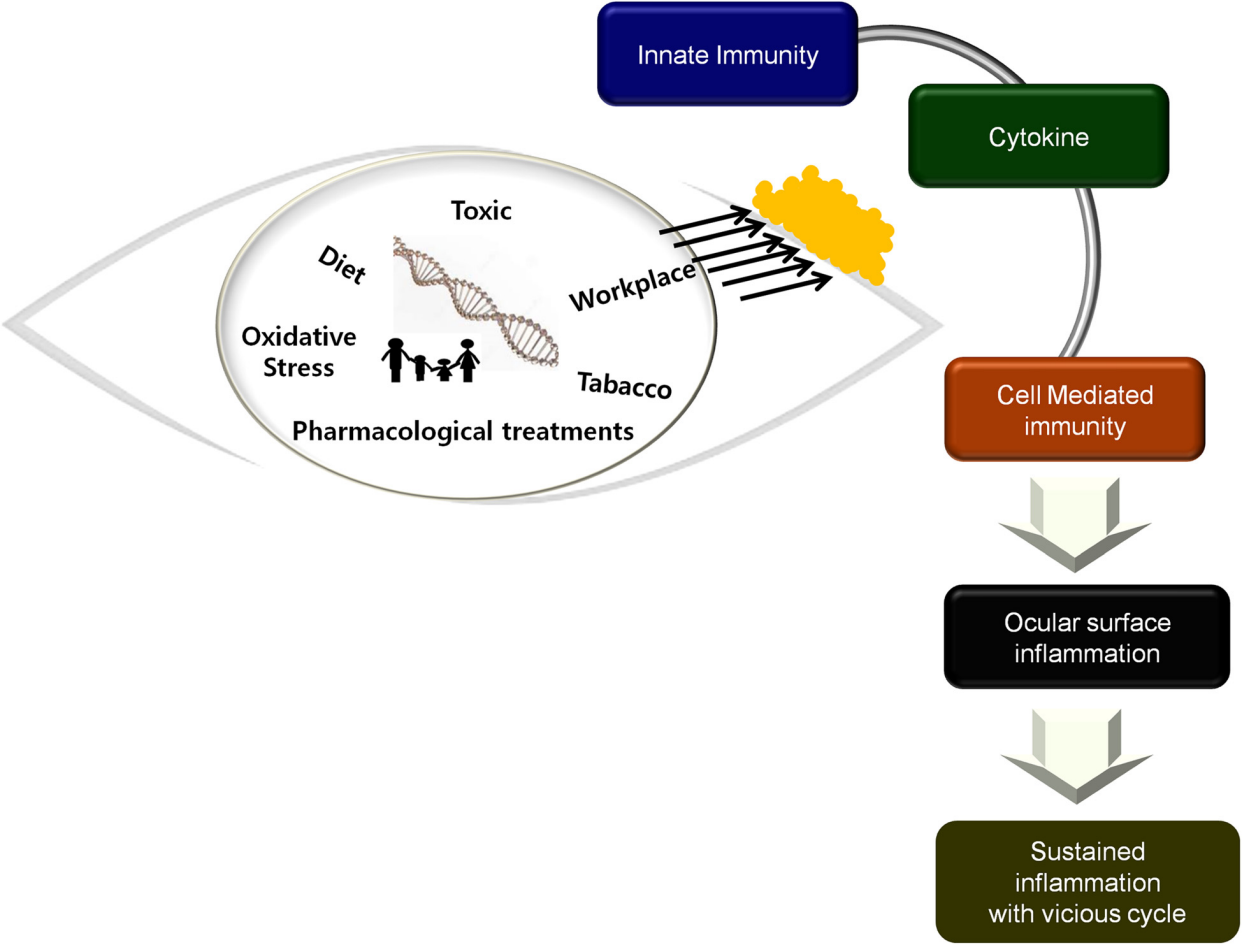
Diagram describing the spiral progression of dry eye disease

This review presents the current evidences for the role of innate immunity and an interface between innate and adaptive immunity in early DED (Table 1). Although the triggering factors remain unclear, there is evidence that changes in the defense mechanism at the ocular surface and genetic factors contribute to the pathogenesis of LFU disruption and inflammation in DED (Fig. 1). Environmental, microbial and endogenous stressors, antigen localization, and genetic factors may provide the trigger for an acute inflammatory event that initiates the vicious cycle of chronic inflammatory DED [35].

**Table 1:** Factors associated with innate immunity and triggering into adaptive immunity

Innate defences	blinking, tear film, epithelium, secretory proteins, mucin, Langerhans cell, extracellular DNA, neutophil extracellular trap
Trigger	T-cell activation, proinflammatory cytokines, genetic polymorphisms, inappropriate control of acute inflammation, homeostasis break

Disruptions to the primary ocular barrier (innate immunity) in DED would be expected to allow for pathogen invasion. However, there has been little evidence published to connect microbial keratitis and DED. This may imply that DED patients have stronger innate immunity to ocular surface damage and infection. It is also possible that changes in the expression of TLRs, innate immune molecules (secretory proteins), and mucins in DED may represent a compromised innate immune response. Failure of the innate response could trigger an adaptive immune response. Clinical and basic science research to accurately evaluate innate and adaptive immunity in DED are necessary because none of the hypothesis itself only (innate, adaptive immunity) can definite in the pathogenesis of DED. The diagnosis of DED, especially in its early stages, has been hampered in clinical settings. Future research should focus on techniques to detect the early signs (innate immunity) in DED patients, and to facilitate the resolution of local inflammation at the ocular surface in order to prevent progression to chronic disease (adaptive immunity). To date, western DED researchers (United States, Europe) have focused on inflammation and the immune response, while Asian researchers (Japan, Korea) have focused on the corneal epithelium.

## Conclusion

Within this review, we discussed the early events and defense mechanism of DED. The current knowledge might be the tip of an iceberg and further studies relating pathogenesis must be sought. By understanding the mechanisms of early event of immune dysfunction through basic science and translational research, potential drug targets can be identified.
